# Salivary Gland Pathology in IgG4-Related Disease: A Comprehensive Review

**DOI:** 10.1155/2018/6936727

**Published:** 2018-04-01

**Authors:** Ilaria Puxeddu, Riccardo Capecchi, Filippo Carta, Antonio Gaetano Tavoni, Paola Migliorini, Roberto Puxeddu

**Affiliations:** ^1^Clinical Immunology Unit, Department of Clinical and Experimental Medicine, University of Pisa, Pisa, Italy; ^2^Otorhinolaryngology Unit, Department of Surgery, Azienda Ospedaliero-Universitaria di Cagliari, University of Cagliari, Cagliari, Italy

## Abstract

IgG4-related disease (IgG4-RD) is a rare fibroinflammatory condition that can affect almost any organ, characterized by swollen lesions and often by eosinophilia and elevated serum IgG4 concentrations. The diagnosis of IgG4-RD is a challenging task: in fact, single or multiple organs can be affected and clinical, serological, and histological findings can be heterogeneous. In IgG4-RD, the involvement of salivary glands is observed in 27% to 53% of patients. Several organ-specific conditions, now recognized as different manifestations of IgG4-related sialadenitis (IgG4-RS), were viewed in the past as individual disease entities. The study of salivary glands may sometimes be complex, because of the number of pathological conditions that may affect them, often with overlapping clinical pictures. Integration of different imaging techniques is often required in the case of swelling of salivary glands, even though biopsy remains the gold standard for a definite diagnosis of IgG4-RS. Thus, in this review, we discuss new insights in the pathogenesis of IgG4-RD, focusing on its clinical aspects and the tools that are currently available for a correct differential diagnosis when the salivary glands are involved.

## 1. Introduction

IgG4-related disease (IgG4-RD) is a rare fibroinflammatory condition that can affect almost any organ [1, 2], including the salivary glands, orbital and periorbital tissues, pancreas, retroperitoneum, and lymph nodes [3]. IgG4-RD is characterized by swollen lesions across organs and often by elevated serum IgG4 concentrations. Diagnosis of IgG4-RD is based on a set of clinical, serological, and pathological criteria [4], and the histological picture is critical for diagnosis. In fact, the hallmark features of the disease are tissue fibrosis with a storiform pattern, a diffuse lymphoplasmacytic infiltrate, obliterative phlebitis, abundance of IgG4^+^ plasma cells, and mild to moderate tissue eosinophilia [5]. Presently, the pathophysiological mechanisms underlying IgG4-RD have not yet been fully established. The increase of IgG4 itself appears to be a reactive phenomenon rather than the primary disease driver. It is likely that in IgG4-RD, the interactions between cells of B cell lineage and a novel CD4^+^ SLAMF7^+^ cytotoxic T cells (CTLs) are involved in the processes leading to tissue inflammation and fibrosis [6–8]. These recent evidences have allowed to identify novel and more specific therapeutical approaches in IgG4-RD, such as targeting the B cell lineage, that have so far given promising results.

### 1.1. Pathophysiology of IgG4-RD

In the last decade, several pathophysiological mechanisms, potentially responsible for the development of IgG4-RD, have been described. B cells and plasmablasts seem to play an important role in IgG4-RD, secreting autoantibodies or acting as antigen presenting cells in the expansion of pathogenic T cells [8, 9]. Oligoclonal IgG4-producing plasmablasts are detected in the peripheral blood of IgG4-RD patients [10, 11], and this population of somatically hypermutated B cells has been proposed as disease biomarker. Furthermore, plasmablasts are reduced by immunosuppressive treatment and reemerge during relapse, thus performing as disease activity marker as well [12, 13]. Recent evidences suggest that in addition to B cells, T cells also play a key role in IgG4-RD pathogenesis [6]. CD4^+^ T cells are the most abundant cells in IgG4-RD lesions; given the eosinophil infiltrate and the levels of IgG4 and IgE, a prominent role of T helper cells type 2 (Th2) cells has been proposed. However, Mattoo et al. [10] demonstrated that relative increases in circulating Th2 were only observed in a subset of patients with IgG4-RD who had a history of atopic disease, while nonatopic IgG4-RD subjects did not exhibit any expansions of circulating Th2 cells. In a more recent study, they also showed clonal expansions of CD4^+^ CTLs in the blood of patients with IgG4-RD [7]. By using multicolor immunofluorescence staining of affected organs, they demonstrated that these CD4^+^ CTLs infiltrated tissue lesions and were the dominant CD4^+^ T cells at disease sites, while CD4^+^GATA3^+^ Th2 cells were sparse [7]. This clonally expanded population of CD4^+^ CTLs, detected in both peripheral blood and fibrotic lesions of IgG4-RD patients, seems to actively contribute to the disease process, particularly to tissue injury and fibrosis. CD4^+^ CTLs, bearing SLAMF7 on their surface, might be actively involved in the fibrotic processes in IgG4-RD, releasing profibrotic mediators such as IL-1*β*, TGF-*β*, and INF-*γ* [7, 11]. To a certain degree, B cell depletion has the potential to attenuate fibrosis associated with IgG4-RD by reducing collagen deposition and myofibroblast activation [14]. In addition, the blood concentration of the CD4^+^SLAMF7^+^ CTLs slowly declines following B cell depletion in IgG4-RD patients, suggesting a direct link between B cells lineage and CD4^+^SLAMF7^+^ CTLs in the pathogenetic mechanisms of IgG4-RD. However, these cells do not express CD20 on their surface and thus are not a target of anti-CD20 treatment, suggesting that CD4^+^ CTLs are sustained by B cells and plasmablasts.

Recently, a role of T follicular helper (Tfh) cells in IgG4-RD pathogenesis has also been proposed [15–17]. Higher proportions of T regulatory and Tfh cells have been detected in IgG4-RD patients compared to healthy controls, correlated with plasmablasts and serum IgG levels [17]. In support to these findings, an increased number and frequency of circulating PD-1^high^ Tfh cells have been detected in peripheral blood of patients, functionally effective in driving IgG4 production from autologous B cells and responsive to steroid treatment [15, 16]. A diffuse infiltrate of Tfh expressing PD-1, ICOS, and BCL6 at high density has been described in tissue lesions [16]. On the whole, these data suggest the possibility to use also circulating Tfh cells as biomarker of disease activity.

Moreover, these new data on T cell subpopulations suggest novel pathogenetic mechanisms and innovative therapeutic approaches to IgG4-RD.

### 1.2. General Clinical Aspects of IgG4-RD

IgG4-RD mainly involves middle-aged to elderly males, unlike classic autoimmune diseases such as systemic lupus erythematosus and Sjogren's syndrome (SSj) that mostly affect females. IgG4-RD occurs in a subacute form in most patients, without the rapid onset of general symptoms such as fever. A minority of patients have weight loss, dramatic elevations of acute phase markers, and other manifestations of systemic inflammation. IgG4-RD typically comes to medical attention because of single-organ involvement, but more widespread disease is often observed following an accurate work-up [18]. Involvement of different organs can occur either simultaneously or metachronously, with the emergence of one newly affected organ following another. IgG4-RD can affect almost any organ, more frequently the salivary glands and pancreas, then the lacrimal glands, lymph nodes, biliary tract and gallbladder, retroperitoneum, thyroid, kidney, lung, periorbital tissues, aorta, and liver [3]. Other organs can also be involved, even if with lower frequency, such as the pituitary gland, meninges, prostate, breast, skin, pericardium, aortic valve, upper airways, ear, pleura, mediastinum, paranasal sinuses, and peripheral nerves.

### 1.3. Diagnosis of IgG4-RD

The diagnosis of IgG4-RD is a challenging task: in fact, single or multiple organs can be affected and clinical, serological, and histological findings can be heterogeneous. IgG4-RD can be suspected in the presence of swollen lesions or diffuse/localized swelling in one or more organs. The diagnostic work-up involves laboratory investigations and imaging such as ultrasonography, computerized tomography (CT) scan, magnetic resonance imaging (MRI), and positron emission tomography (PET). Histopathology is mandatory for diagnostic purposes and also to exclude neoplastic or other inflammatory disorders [19]. Criteria for the diagnosis of IgG4-RD have been proposed by Umehara et al. [4], as summarized in Table 1. A definite diagnosis is made only when three criteria are met: evidence of diffuse/localized swelling or mass lesions in one or more organs, elevated serum IgG4 concentrations, and a marked lymphoplasmacytic infiltration and fibrosis with IgG4^+^ plasma cells at histology. When serological criteria (e.g., increased serum IgG4 levels) are not fulfilled, the disease is considered “probable” and “possible” when only the clinical and serological criteria are met. Less stringent criteria are used in the case of type I autoimmune pancreatitis (AIP) and Mikulicz's disease (MD): in these localizations, histological data are not essential [4]. Differential diagnosis encompasses benign and malignant tumors, especially lymphomas, and disorders with a similar clinical picture: SSj, Castleman disease, sarcoidosis, granulomatous polyangiitis, primary sclerosing cholangitis, retroperitoneal fibrosis, and eosinophilic granulomatosis with poliangiitis. Differential diagnosis from a neoplastic disorder is particularly difficult when the disease affects a single organ or represents an accidental finding of a radiological or histological test. On the other hand, when IgG4-RD is multiorgan, lymphoma or a metastatic disease should be excluded; the clinical picture is often confusing, as both weight loss and lymphadenopathy can be present in all these conditions. Serology can be more helpful, if eosinophilia and hypergammaglobulinemia with increased serum levels of IgE and especially of IgG4 are detected. An increased number of IgG4^+^ plasma cells in tissues are a more specific finding, but not exclusive of IgG4-RD. It is in fact observed in ANCA-associated vasculitis and urticarial vasculitis, in hematological malignancies, in pancreatic or lung cancer and sarcoma, in tumor of salivary glands and lymphoma, in infections, in inflammatory bowel diseases and diverticulitis, and in rheumatoid arthritis and histiocytosis. However, the other typical hystopathological findings of the disease are not present in any of these disorders. Therefore, histology remains, up to now, mandatory for differential diagnosis; clinicopathological correlation is also essential.

### 1.4. Therapy of IgG4-RD

In a few cases, spontaneous remission of the disease has been reported and “watchful waiting” represents an option in case of involvement limited to submandibular glands and lymph nodes. When vital organs are affected, or the disease has an aggressive course, treatment is necessary to prevent organ dysfunction. IgG4-RD has a significant response to treatment with immunosuppressants. Steroids represent the cornerstone of treatment. It has been shown that steroid treatment improves the function of affected organs, prevents organ damage, and decreases the rate of recurrence [20]. Response to treatment is observed within 2 weeks, with disappearance of symptoms, decrease of serum IgG4 levels, and improvement of organ function. Prednisolone is usually employed, at the initial dose of 0.6 mg/kg for 2–4 weeks, with gradual tapering (5 mg every week/2 weeks) [1]. Steroid treatment can be interrupted in 3–6 months, or a low dosage (2.5–5 mg/day) can be maintained for 3 years, as suggested by Japanese authors.

Steroid-sparing agents are often employed, as in other autoimmune disorders, to maintain disease control with lower steroid dosage or without steroids. Azathioprine, methotrexate, and mycophenolate mofetil have all been used, and less often 6-mercaptopurine and cyclophosphamide [21].

In patients with recurrent or refractory disease, the monoclonal anti-CD20 Rituximab has been proven to be effective, inducing a decrease in serum IgG4 levels and a rapid clinical response. The parallel decrease in CD20^+^ B cells and serum IgG4 levels suggests that IgG4 are mainly produced by short-lived plasmablasts and plasma cells, rapidly depleted by a treatment affecting mature B cells. Rituximab therapy reduces inflammatory infiltrate and also, albeit partially, fibrosis [21].

### 1.5. Salivary Gland Involvement in IgG4-RD: General Aspects

In IgG4-RD, the involvement of salivary glands is observed in 27% to 53% of the patients [22]. Several organ-specific conditions, now recognized as different manifestations of IgG4-related sialadenitis (IgG4-RS) [23], were viewed in the past as individual disease entities. For example, MD, a dramatic bilateral painless swelling of parotid, lacrimal, and submandibular glands, was previously linked and not clearly distinguished from SSj until 2005, because of their similar glandular histological aspects [24]. Similarly, the Kuttner's tumor or chronic sclerosing sialadenitis, characterized by severe swelling of the submandibular glands, was initially considered as an individual disease entity, frequently associated with sclerosing cholangitis and retroperitoneum involvement.

#### 1.5.1. Salivary Gland Involvement in IgG4-RD: Clinical Presentation

In IgG4-RS, the swelling of lacrimal and salivary glands is mostly, but not exclusively, bilateral and painless and persists generally for more than 3 months [25]. Submandibular glands are more frequently affected, but parotid, sublingual, and labial salivary glands are also involved. Usually, salivary secretion is normal or slightly reduced and xerostomia is present in 30% of the patients, less frequently than in SSj. The secretory impairment, when present, is more severe in submandibular glands [23] and improves with an early steroid treatment. It has been reported that in 40% of patients affected by type I autoimmune pancreatitis (AIP), IgG4-RS is also present. IgG4-RD patients with salivary involvement can experience more frequently AIP, sclerosing cholangitis, and asthma compared to those with SSj [23, 26]. Along with the salivary and lacrimal glands, other otorhinolaryngological sites may be involved in IgG4-RD, such as the nose, paranasal sinuses, and ears, with a frequency higher than 50% [27]. Furthermore, cervical lymphadenopathy can be found in 70% of IgG4-RS patients, raising the suspicion of an underlying IgG4-RS in subjects with enlarged salivary glands [23].

In our cohort of 20 IgG4-RD patients (M : F ratio 9 : 11, mean age 59, range 18–81), salivary gland involvement was observed in 3 cases. Diagnosis of IgG4-RD, according to the above described criteria [4], was definitive in all 3 patients. One patient was affected by MD with dacryoadenitis and parotid involvement with cervical lymphoadenopathy; symptoms were xerostomia, swelling, and oedema of parotid glands. Serological (elevated serum IgG4, 388 mg/dL) and histological data (biopsy of parotid with storiform fibrosis, lymphomonocytic infiltrate with high quote of IgG4^+^ plasmablasts) led to the diagnosis.

**Figure 1:**
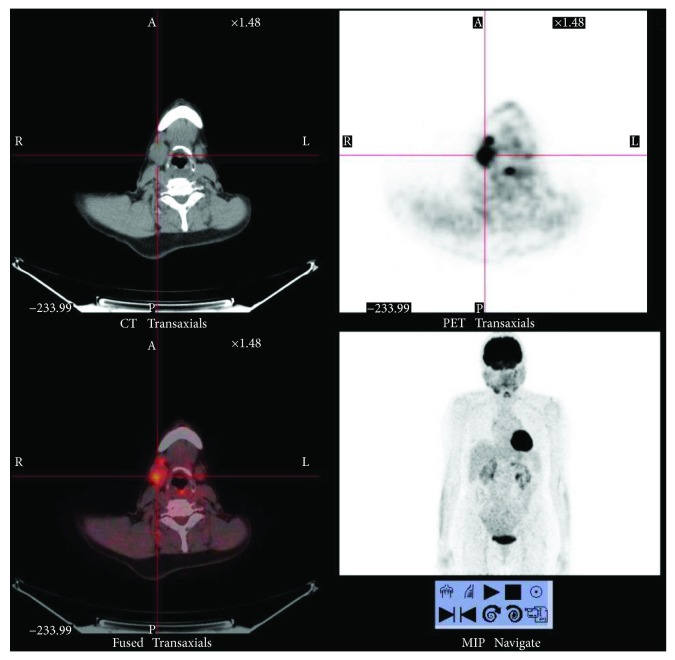
Positron emission tomography (PET) in an IgG4-RD patient with dacryoadenitis and sialoadenitis. PET analysis was performed during relapse of the disease before starting new therapy. In the right submandibulary region and pretracheal lymph nodes, the PET analysis shows an enlargement of the parotid gland with an increase concentration of 18-FDG and glucidic hypermetabolism.

The other two patients were affected by both MD and Kuttner's tumor, with AIP and retroperitoneal fibrosis. Their clinical picture was characterized by longer disease duration (17 and 35 years, resp.), higher serum IgG4 titer (1870 mg/dL and 304 mg/dL at the diagnosis), and more frequent relapses of the disease. In one patient, an enlargement of the parotid was detected by PET analysis (Figure 1); in the other one, the submandibular gland involvement was recognized retrospectively after gallium-67 scintigraphy in the absence of any local symptom, such as xerostomy.

#### 1.5.2. Salivary Gland Involvement in IgG4-RD: Differential Diagnosis

In the presence of a mass lesion of salivary glands, the key point is to exclude a tumor. Differential diagnosis involves salivary gland neoplasms and other malignancies such as lymphoma or metastatic tumors. As an IgG4-producing marginal zone B cell lymphoma was described, to exclude a lymphoma may not always be easy [28]. Other entities that can mimic an IgG4-RS include SSj, Castleman disease, eosinophilic granulomatosis with poliangiitis, sarcoidosis, and the Heerfordt syndrome, characterized by extrapulmonary manifestations, in which salivary glands and cervical lymph nodes are involved, and uveitis and facial nerve palsy can be present. Among the various diseases to be taken into account for a differential diagnosis with IgG4-RD, SSj can often be challenging. These two diseases often share common clinical and laboratory aspects, such as glandular enlargement, sometimes sicca symptoms, arthralgias, hypergammaglobulinemia, hypocomplementemia, and the presence of antinuclear antibodies (ANA). However, there are features that distinguish the two entities, for example, the presence of anti-Ro/SSA and anti-La/SSB antibodies in the vast majority of SSj patients, the IgG4^+^ plasma cells infiltration, and the response to steroids in IgG4-RD. However, in a few cases, the two disorders can coexist. Nakashima et al. [29] described a case in which the diagnostic criteria of both IgG4-RD and SSj were met. Furthermore, Baer et al. [30] reported that only one out of 2594 patients in a research registry for SSj presented histopathological findings consistent with the diagnosis of IgG4-RD. In a study including 133 patients with primary SSj, Mavragani et al. [31] described circulating IgG4 levels higher than 135 mg/dL in 10 patients and a marked infiltration of IgG4^+^ plasma cells in the minor salivary glands of 3 patients. On the other hand, Yamamoto et al. [32] described 7 patients out of 160 affected by MD that were positive for anti-Ro/SSA antibodies and met the American College Rheumatology criteria and the American European consensus criteria for SSj. However, the use of low stringency diagnostic criteria for MD, criteria that are presently under revision, suggests a potential misdiagnosis in some of these patients. In conclusion, coexistence of the two disorders can be suspected in a very limited number of patients, but an “IgG4-RD/SSj overlap” has not so far been proposed.

#### 1.5.3. Salivary Gland Involvement in IgG4-RD: Imaging and Diagnostic Techniques

The study of salivary glands may sometimes be complex, because of the number of pathological conditions that may affect them, often with overlapping clinical pictures. Integration of different imaging techniques is often required in the case of swelling of salivary glands, even though biopsy remains the gold standard for a definite diagnosis of IgG4-RS. Among the different methods of imaging useful for the study of salivary glands, ultrasonography represents the most widely used technique. This is mainly due to its noninvasiveness and high tolerability for the patient, as well as for the low cost. Even if some ultrasonography features can be shared by IgG4-RS and SSj [33], the recent introduction of the color Doppler has allowed to reveal typical features of IgG4-RS in salivary glands, such as increased color Doppler signaling ratios [34]. Intraductally applied contrast-enhanced ultrasound (IA-CEUS) shows also promising results to depict the changes of the parenchyma of the gland due to incomplete contrast filling resulting from numerous small cysts [35].

PET is an emerging diagnostic option in the context of IgG4-RD, since organ lesions accumulate 18F-fludeoxyglucose at high concentration. Although this technology does not allow a specific distinction between inflammatory and cancerous lesions, it can be very useful in the identification of organ involvement beyond the salivary glands, such as the pancreas, retroperitoneum, and periaortic tissue, that can be affected by IgG4-RD, even if clinically silent. Moreover, this technique could be a tool for targeting specific biopsy sites, monitoring disease activity, and also evaluating response to treatment [36]. However, PET is an expensive diagnostic tool and its use must always be targeted.

Even if CT scan and MRI are useful techniques in the diagnosis of salivary glands swelling, they have some limitations in IgG4-RS diagnosis. Recently, Shimizu et al. [33] have conducted a study on the effectiveness of various imaging modalities in the screening of IgG4-RS, focusing on the differences with SSj and on the detection of typical features. They conclude that the nodal changes in IgG4-RS detected by ultrasonography were not clearly observed on CT scan or MRI and that ultrasonography, but not CT scan and MRI, is an effective imaging modality to differentiate IgG4-RS from SSj. IgG4-RS may have characteristics similar not only to SSj but also to chronic obstructive submandibular sialadenitis, one of the most common disorders of submandibular glands, characterized by the obstruction of the ductal system by various causes. Yamamoto et al. [37], comparing the three conditions, observed that sialography was effective to differentiate IgG4-RS from SSj, but not from chronic obstructive submandibular sialadenitis. A rising interest is on the potential role of sialendoscopy in salivary gland pathology that allows a precise evaluation of the duct system avoiding sialography. Sialendoscopy represents a promising gland-preserving tool in the management of nonstone disorders of major salivary glands [38].

Biopsy remains mandatory for establishing a diagnosis and should be analyzed as described above. However, slight differences in the histological pattern can be seen in tissue sample from labial salivary glands, parotid glands, or submandibular glands. For example, an intense tissue fibrosis seems to be a common feature of biopsies obtained from submandibular glands [39, 40], and obliterative phlebitis is present in almost one-third of patients with submandibular involvement. Conversely, both characteristics are rare in tissue samples from labial salivary glands. Labial salivary gland biopsy is relatively easy to obtain, but its sensitivity for the diagnosis of IgG4-RD is very low [40, 41]. Involvement of parotid and submandibular glands, on the other hand, leads to more destructive surgical interventions. Deshpande et al. [5] introduced the following diagnostic cut-off in the sample biopsies, more than 30–50 IgG4^+^ plasmacytes per high-power field and a ratio of IgG4^+^ to IgG^+^ cells greater than 40%. However, a tissue-specific cut-off for tissue IgG4^+^ plasma cells in IgG4-RD has not yet been validated. Ectopic germinative centers and occasional eosinophilic infiltration in the affected tissue are common findings. Given the recent description of a case of marginal zone B cell lymphoma that produced IgG4, Takano et al. [42] recommend to perform Western blot analysis of immunoglobulin heavy-chain gene rearrangement. Furthermore, they also propose to perform biopsy of submandibular glands as a “surrogate biopsy” for the diagnosis of AIP. They describe 10 patients with AIP and submandibular biopsies diagnostic for IgG4-RD, but only in 4 patients the biopsy of minor labial glands fulfilled the diagnostic criteria for IgG4-RD [43]. In line with these results, we observed that in 3 IgG4-RD patients with pancreatic involvement, the labial salivary gland biopsy was not diagnostic for IgG4-RD, confirming that this procedure has an insufficient diagnostic sensitivity.

### 1.6. Conclusion and Perspectives

IgG4-RD is a multisystem disease that can sometimes involve single organs such as the lacrimal and salivary glands. When the disease is suspected, the two key points are as follows: (1) the differential diagnosis from solid tumors and lymphomas (especially in the presence of mass lesions) and (2) the study of multiple organ localizations (by means of imaging techniques). Biopsy of affected organs remains so far the gold standard for diagnosis. Therapy is presently based on the use of steroids and immunosuppressants, but recent insights into pathogenic mechanisms forecast new more disease-tailored therapeutic approaches.

## Figures and Tables

**Table 1:** Diagnostic criteria for IgG4-related disease (modified after Umehara et al. [4]).

Diagnosis	Criteria
Definitive	Diffuse or local swelling or multiple organs
	Serum IgG4 levels ≥ 135 mg/dL
	Histology:
	(1) Lymphoplasmacytic infiltrate and fibrosis (2) IgG4^+^ plasma cells: ratio of IgG4^+^/IgG^+^ cells > 40%, and >10 IgG4^+^ plasma cells/high-power field

Probable	Diffuse or local swelling or multiple organs
	Histology:
	(1) Lymphoplasmacytic infiltrate and fibrosis (2) IgG4^+^ plasma cells: ratio of IgG4^+^/IgG^+^ cells > 40%, and >10 IgG4^+^ plasma cells/high-power field

Possible	Diffuse or local swelling or multiple organs
	Serum IgG4 levels ≥ 135 mg/dL
